# Analysis of hedgehog signaling in periocular sebaceous carcinoma

**DOI:** 10.1007/s00417-018-3900-5

**Published:** 2018-02-08

**Authors:** John C. Bladen, Mariya Moosajee, Dhani Tracey-White, Michèle Beaconsfield, Edel A. O’Toole, Michael P. Philpott

**Affiliations:** 1 Eyelid Oncology, Moorfields Eye Hospital, London, UK; 2 Centre for Cell Biology and Cutaneous Research, Blizard Institute, Barts & London School of Medicine, 4 Newark St, London, E1 2AT UK; 3 Department of Ocular Biology and Therapeutics, UCL Institute of Ophthalmology, London, UK

**Keywords:** Hedgehog pathway, Sebaceous carcinoma, Stroma, Immunofluorescence

## Abstract

**Purpose:**

Sebaceous carcinoma (SC) is a clinical masquerader of benign conditions resulting in significant eye morbidity, sometimes leading to extensive surgical treatment including exenteration, and even mortality. Little is known about the genetic or molecular basis of SC. This study identifies the involvement of Hedgehog (Hh) signaling in periocular SC.

**Methods:**

Fifteen patients with periocular SC patients were compared to 15 patients with eyelid nodular basal cell carcinoma (nBCC; a known Hh tumor), alongside four normal individuals as a control for physiological Hh expression. Expression of Patched 1 (PTCH1), Smoothened (SMO), and glioma-associated zinc transcription factors (Gli1 and Gli2) were assessed in histological sections using immunohistochemistry and immunofluorescence (IF) techniques. Antibody specificity was verified using Western-blot analysis of a Gli1 over-expressed cancer cell line, LNCaP-Gli1. Semi-quantification compared tumors and control tissue using IF analysis by ImageJ software.

**Results:**

Expression of the Hh pathway was observed in SC for all four major components of the pathway. PTCH1, SMO, and Gli2 were more significantly upregulated in SC (*P* < 0.01) compared to nBCC. Stromal expression of PTCH1 and Gli2 was observed in SC (*P* < 0.01). In contrast, stromal expression of these proteins in nBCC was similar or down-regulated compared to physiological Hh controls.

**Conclusions:**

The Hh signaling pathway is significantly more upregulated in periocular SC compared to nBCC, a known aberrant Hh pathway tumor. Furthermore, the stroma of the SC demonstrated Hh upregulation, in particular Gli2, compared to nBCC. Targeting of this pathway may be a potential treatment strategy for SC.

**Electronic supplementary material:**

The online version of this article (10.1007/s00417-018-3900-5) contains supplementary material, which is available to authorized users.

## Introduction

Sebaceous carcinoma (SC) is a rare, aggressive cancer that has a predilection for the periorbital region, but can originate from extraocular sites, albeit mainly within the head region [1]. Geographical variation is significant, with the incidence around 0.41 per million in UK, 0.65 per 100,000 in Canada, whereas in China it represents almost one-third of the malignant eyelid workload and is second to basal cell carcinoma (BCC) in frequency [2–4]. In Japan, one study found that the rate of SC equaled that of BCC [5]. SC has two broad macroscopic presentations, namely nodular (local) or pagetoid (spreading). Misdiagnosis is common, often labeled as a benign chalazion in its nodular form or conjunctivitis in the conjunctival, pagetoid type (Supplementary Fig. S1). Thus, it is described as a masquerading lesion with delays in diagnosis often greater than a year. Pagetoid expansion of the skin or conjunctiva carries a higher risk of orbital exenteration [6]. Despite aggressive treatment, there is a high recurrence rate and metastasis often occurs via the lymphatics to the cervical lymph nodes [7]. Mortality rates were once very high at 29%, but there is a significant range depending on which treatment modality is employed and can be as good as 6% in a center of excellence [8–10]. Prognosis depends on the size of the initial tumor and margin removed with a higher risk of recurrence seen with 1-3 mm margin versus 5 mm; however, achieving such a margin if the tumor is within the orbit is almost impossible [11, 12]. Staged excision using margin control may also improve removal of the tumor.

Hedgehog (Hh) signaling is essential during embryogenesis, and is down-regulated in most adult cells with transient expression in some adult tissues including brain, testis, and hair follicle [13, 14]. Hh signaling occurs via a cell membrane receptor complex that includes the proteins Patched (PTCH1) and Smoothened (SMO). In the absence of a Hh signal, PTCH1 inhibits the activation of SMO and subsequent downstream signaling. In the presence of Hh, the repressive action of PTCH1 on SMO is removed and the downstream pathway is mediated via the glioma-associated zinc transcription factors, Gli1 and Gli2, which translocate to the nucleus and activate Hh-target genes [15]. Pathological activation of Hh signaling occurs in a variety of cancers, including BCC, prostate cancer, upper gastrointestinal tumors, a subset of small cell lung carcinoma and pancreatic ductal adenocarcinoma (Supplementary Table S1) [16–20]. Genetic variants in Hh signaling are seen in up to 90% of sporadic BCC and many demonstrate Gli1 overexpression [19, 21].

The behavior of solid tumors is not restricted to the tumor borders delineated on microscopy; the surrounding non-malignant microenvironment, termed stroma, has been shown to play a key role in local spread [22]. During embryogenesis, Hh signaling plays an important role in communication between epithelial tissue and surrounding stroma in a number of tissues in order to promote growth and differentiation of the stroma. For instance, urogenital epithelium utilizes Hh signaling to promote surrounding mesenchymal tissue to develop into the prostate gland [23]. Reactivation of this paracrine phenomenon of Hh signaling has been shown in prostate cancer stroma and the tumor regulates the proliferation of adjacent non-malignant epithelium [24]. Studies in ovarian cancer have highlighted the importance of the changes in the stroma that confer a poorer prognosis [22, 25] and the promotion of local invasion in morphoeic BCC has been demonstrated by modulating the stromal milieu [26]. In this study, we examined the role of the canonical Hh pathway in both the SC tumor and surrounding apparently normal stroma by comparing it to nodular BCC (nBCC), a known Hh implicated tumor.

## Materials and methods

### Subjects

This study obtained national ethics committee approval (REC reference 14/NW/1080) and adhered to the tenets of the Declaration of Helsinki. The research was designed as a non-paired observational, case control study whereby SC (the case) is compared to nBCC (the Hh tumor control). Fifteen patients with SC were included for comparison against 15 nBCC patients with a mean age of 68 and 73, years respectively. All of the SC patients had non-metastatic disease, ten nodular and five pagetoid, requiring wide excision or exenteration as treatment. Only clearly defined SC or BCC on histology were included using the Royal College of Pathologists, UK dataset [27, 28]. The presence of any non-nodular BCC subtype, and poorly differentiated SC where it was difficult to identify adnexal sebaceous lineage, were also excluded from the study. In addition, exclusion criteria included SC or BCC individuals with metastasis and/or recurrence. Subjects were seen at one center, Moorfields Eye Hospital.

### Immunostaining procedure

Hematoxylin and eosin (H&E) staining was performed using standard methods. 3–3′-diaminobenzidine (DAB) immunostaining of 5 μm formalin-fixed, paraffin-embedded (FFPE) tissue sections was performed using a panel of commercially available antibodies for the Hh pathway proteins including PTCH1, SMO, Gli1, and Gli2 (Abcam, Cambridge, UK). Conditions for individual antibodies are summarized in Supplementary Table S2. Tissue sections were mounted on slides, deparaffinized in xylene and then alcohol, followed by treatment with the DAKO EnVision™FLEX+ System (Dako, Glostrup, Denmark) [29]. Briefly, antigen retrieval was performed using the DAKO PT LINK machine at 97°C for 20 min under alkaline (pH 9.0) or acidic (pH 6.0) conditions (Table S1). Endogenous peroxidase activity was blocked using EnVision™FLEX peroxidase-blocking reagent. Tissue was incubated with primary antibody for 30 min, followed by incubation with a secondary antibody (Envision™FLEX/HRP LINKER; 20 min) and then visualization of the primary-secondary reaction with EnVision™FLEX substrate working solution containing buffer and DAB (10 min). Sections were sealed and covered using the automated Thermo Clearview Coverslipper (Thermo Fisher Scientific Inc., Waltham, MA, USA). Positive control tissue included breast, brain, testes, and intestine for PTCH1, SMO, Gli1, and Gli2, respectively. Negative controls involved all steps excluding the primary antibody. Finally, sections were counterstained with hematoxylin using the Gemini autostainer (Thermo Fisher Scientific Inc., Waltham, MA, USA).

### Immunofluorescence

Immunofluorescence was used for semi-quantitative analysis of Hh pathway expression in SC in comparison to nBCC. FFPE sections were cut (5 μm) and antigen retrieval carried out as described above. Blocking of the sections occurred with 5% goat serum for 1 h followed by primary antibody staining as mentioned. Secondary antibody staining utilized AlexaFluor-568 (Invitrogen™, Life Technologies, Thermo Fisher Scientific Inc., Waltham, MA, USA). Nuclei were counterstained with 4′,6-diamidino-2-phenylindole dihydrochloride (DAPI; Sigma-Aldrich® MO, USA) at 1:1000 dilution to highlight the number of cells present and aid accurate semi-quantification. Coverslips were placed using VECTASHIELD® mounting medium (Vector Laboratories Inc., Burlingame, CA, USA). Negative controls omitted the primary antibody.

### Confocal microscopy and fluorescence signal quantification

Immunofluorescence sections were examined using the Zeiss LSM710 Meta confocal laser microscope (Carl Zeiss Microscopy GmbH, Jena, Germany) and a ZEN configuration tool (Carl Zeiss Microscopy GmbH, Jena, Germany), which is digital image-processing software that produces an output TIFF image. Negative control slides were used to remove background fluorescence and set up the acquisition conditions that subsequently remained constant throughout the imaging process. Images were taken at 200× magnification. Output TIFF images were analyzed using ImageJ (http//imagej.nih.gov/ij) to quantify antibody expression. For semi-quantification of antibody expression, fluorescence intensity was determined in regions of interest (ROIs), ensuring a standardized area size whilst containing the same number of nuclei (averaging 18 cells) as determined by the DAPI staining. Tumor was defined as within the bulk of the tumor and at least 1 mm from the edge. Stroma was defined as non-cancerous surrounding tissue within 2 mm of the tumor edge. Furthermore, three separate ROIs were taken for each area in each sample (i.e., three tumor ROIs and three stroma ROIs in nBCC or SC samples) to obtain a mean signal for comparison.

### Western blotting for antibody validation against Hh pathway

Hh pathway antibodies were validated against protein lysates from LnCaP cells ectopically expressing Gli1 with resulting upregulation of the key effectors of Hh signaling [30]. Negative control tissue was human wild-type fibroblasts. Pre-cast Any kD™SDS-polyacrylamide gel (Bio-Rad, Boston, MA, USA) was used to separate proteins. Twenty micrograms of protein was heated for 5 min at 95°C and mixed with 5X loading dye (100 mM Tris-GCL pH 6.8, 4% SDS; 200 mM dithiothreitol, 0.2% bromophenol blue, and 20% glycerol). Exact volume varied in order to get 20 μg of protein per lane. DualColor Prestained Protein Ladder (Bio-Rad) was used for the identification of protein size. The gel was placed into a mini-PROTEAN tetra cell system (Bio-Rad) and run for 1 h and 30 min at 60 V in 1× Nupage MES SDS running buffer (Life Technologies, Thermo Fisher Scientific Inc., Waltham, MA, USA). Protein was transferred onto an Immun-Blot™ PVDF membrane (Bio-Rad) using a Trans-Blot SD semi-dry transfer cell (Bio-Rad) and Nupage Transfer Buffer (Life Technologies) at ~8 V for 1 h. Subsequently, the membrane was blocked overnight at 4 °C in blocking solution (0.4% PBS/Tween (PBST), 5% dry milk). The following morning, the membrane was washed five times in PBST and then incubated for 2 h at room temperature with the antibody of interest (1:500 anti-PTCH, 1:200 anti-SMO, 1:1000 anti-Gli1, and 1:500 anti-Gli2). The membrane was then washed five times in PBST for 5 min, and incubated with 1:10,000 conjugated secondary anti-rabbit IgG horseradish peroxidase (HRP) (Dako, Glostrup, Denmark) for 1 h at room temperature. A further five washes of PBST for 5 min before the use of an ECL™ Prime Western blotting detection system kit (GE Healthcare, Buckinghamshire, UK). All blots were imaged under the same conditions; signal was detected using the ChemiDoc MP Imaging system (Bio-Rad) and ImageLab software (Bio-Rad). Membranes were stripped of primary antibody using Restore™ Western Blot Stripping Buffer (Thermo Fisher Scientific, Inc) at 55 °C for 30 min, washed five times for 5 min in PBST, and reprobed with 1:5000 polyclonal anti-β-actin antibody (Sigma-Aldrich, MO, USA) as a loading control for each sample.

### Statistical analysis

Recorded immunofluorescence signal data for each sample is expressed as a mean ± SEM. Comparison between SC, nBCC, and normal groups was made using the Mann–Whitney* U* test. Critical values were identified for a *p* value of less than 0.05 and 0.01 and a* U* value less than these were considered to be significant.

## Results

**Fig. 1:**
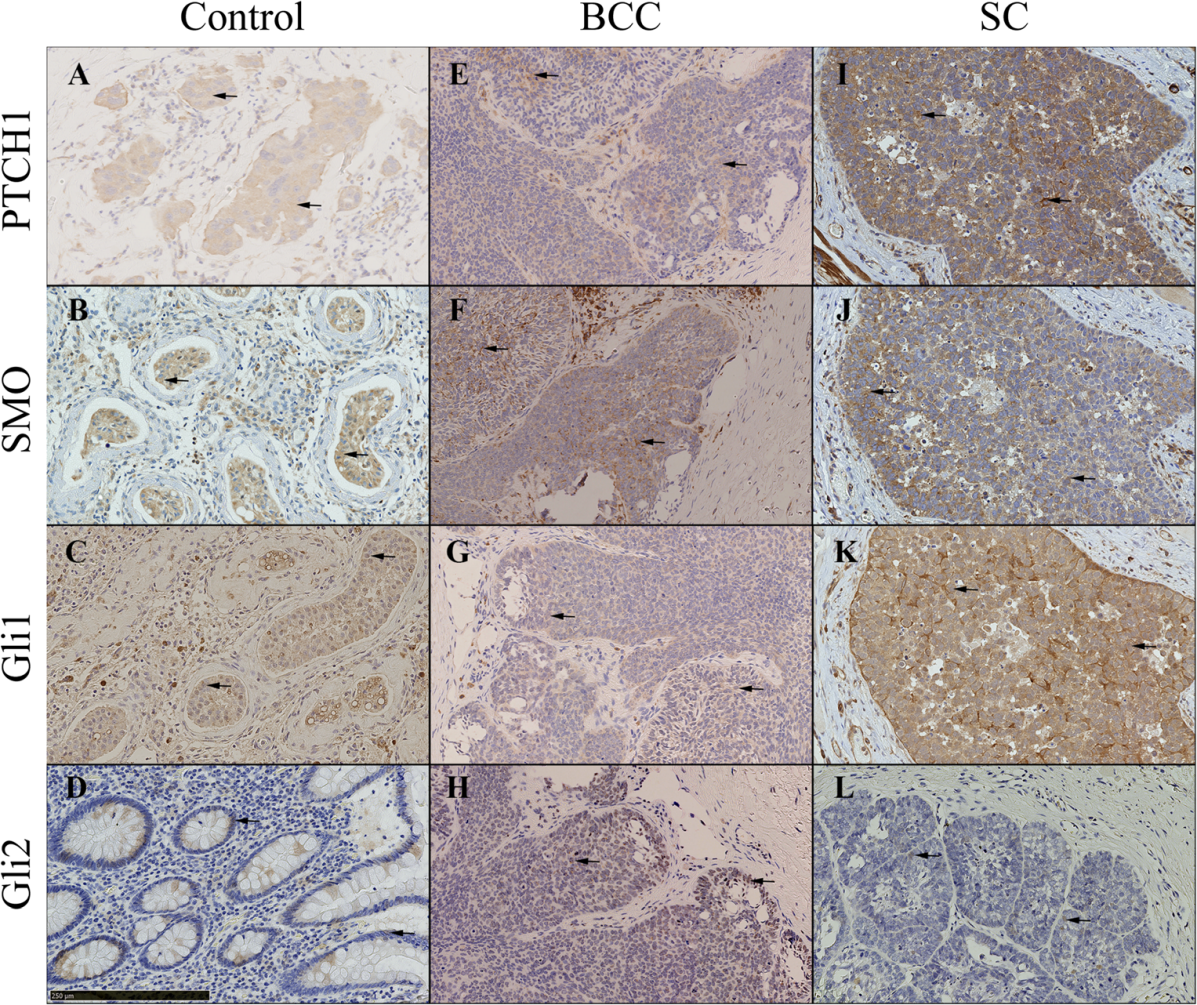
Representative pictures of Hedgehog pathway expression in positive control tissue (**a**-**d**), nBCC (**e**-**h**) and SC (**i**-**l**). **a** Breast tissue displays PTCH1 expression in the cytoplasm of the cells (*black arrows*). **b** SMO expression in testicular tissue, within the Leydig cells and seminiferous tubules cells (*black arrows*). **c** Gli1 nuclear and cytoplasmic expression in testicular tissue, particularly in the Leydig cells and seminiferous tubules (*black arrows*). **d** Nuclear expression of Gli2 in intestinal tissue, specifically localized to epithelial cells of the intestinal glands. **e** PTCH1 expression in the cytoplasm (*black arrows*) of nBCC, similar to control tissue. **f** Similar SMO expression in nBCC compared to control. **g** Increased Gli1 nuclear expression in nBCC compared to control tissue. **h** Increased Gli2 nuclear expression compared to control tissue. **i** Marked PTCH1 cytoplasmic expression in SC compared to control and nBCC. **j** Slight increase in staining of SMO in the cytoplasm (*black arrows*) of SC in contrast to nBCC. **k** Increased staining of nuclear (*black arrows*) and cytoplasmic Gli1 in SC compared to nBCC, and increased cytoplasmic expression compared to control. **l** Nuclear staining of GLi2 (*black arrows*) in SC that is similar to nBCC, but more than control tissue.* Scale bar* represents 250 μm and all images are at 200× magnification. Antibody stains for PTCH1 (**a**, **e**, **i**), SMO (**b**, **f**, **j**), Gli1 (**c**, **g**, **k**) and Gli2 (**d**, **h**, **l**).* PTCH1* Patched 1,* SMO* Smoothened,* Gli1* glioma-associated zinc transcription factor1,* Gli2* glioma-associated zinc transcription factor2

**Fig. 2:**
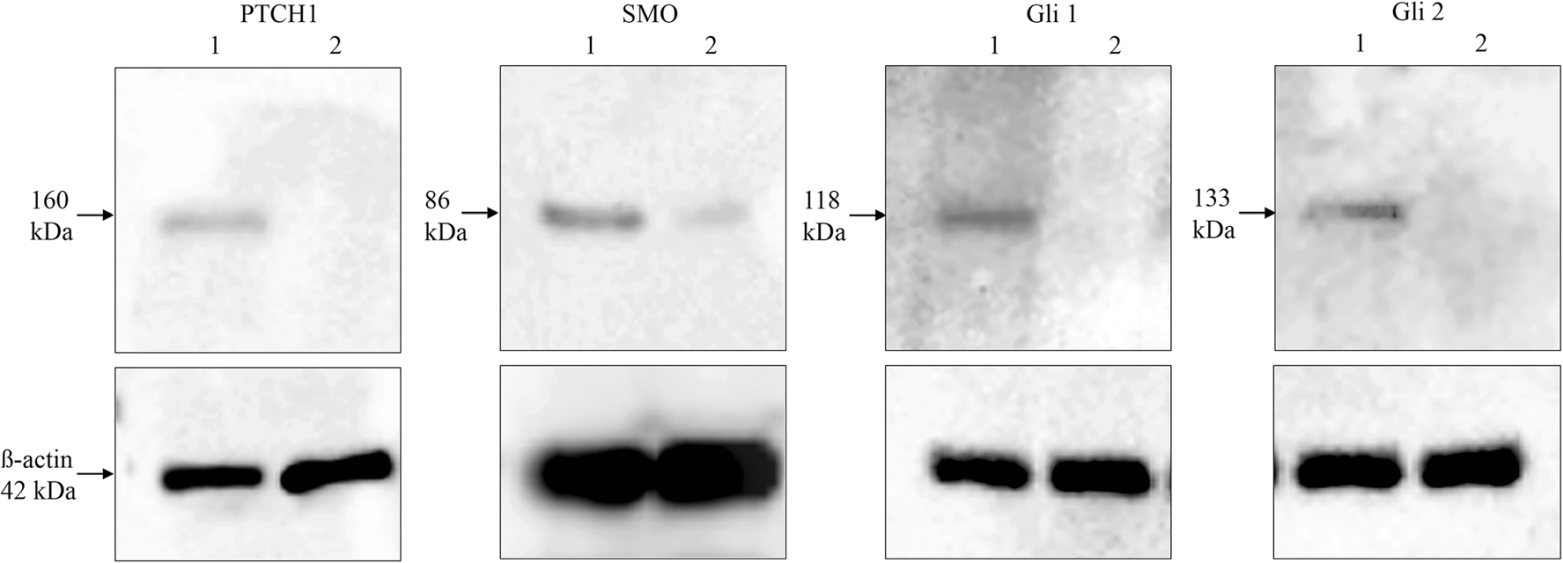
Western-blot analysis of LnCaP-Gli1 and human wild-type fibroblasts demonstrating good specificity of each antibody with a clear band for the appropriate sized protein (20 μg per lane, *n* = 3 biological replicates). 1 = LnCaP-Gli1; 2 = Human wild-type fibroblasts.* PTCH1* Patched 1,* SMO* Smoothened,* Gli1* glioma-associated zinc transcription factor1,* Gli2* glioma-associated zinc transcription factor2

Hh pathway expression was detected in all 15 SC and nBCC tumors as demonstrated by the DAB immunostaining for PTCH1, SMO, Gli1, and Gli2 (Fig. 1). Western blot of LNCaP-Gli1 cells, a known hyper-expressed Hh pathway cancer cell line, demonstrated good specificity of each antibody with a clear band for the appropriate-sized protein (Fig. 2). PTCH1 expression was detected in the cytoplasm of both nBCC and SC (Fig. 1 e and i, respectively) however, PTCH1 expression was markedly more pronounced in SC compared to nBCC. Similar levels of SMO were observed in both nBCC and SC (Fig. 1f and j). Gli1 and Gli2 were detected in the cytoplasm and nuclei of both nBCC and SC (Fig. 1g, k, h, and i, respectively). Gli1 expression was stronger in SC compared to nBCC, whereas similar levels of Gli2 were observed in both nBCC and SC.

**Fig. 3:**
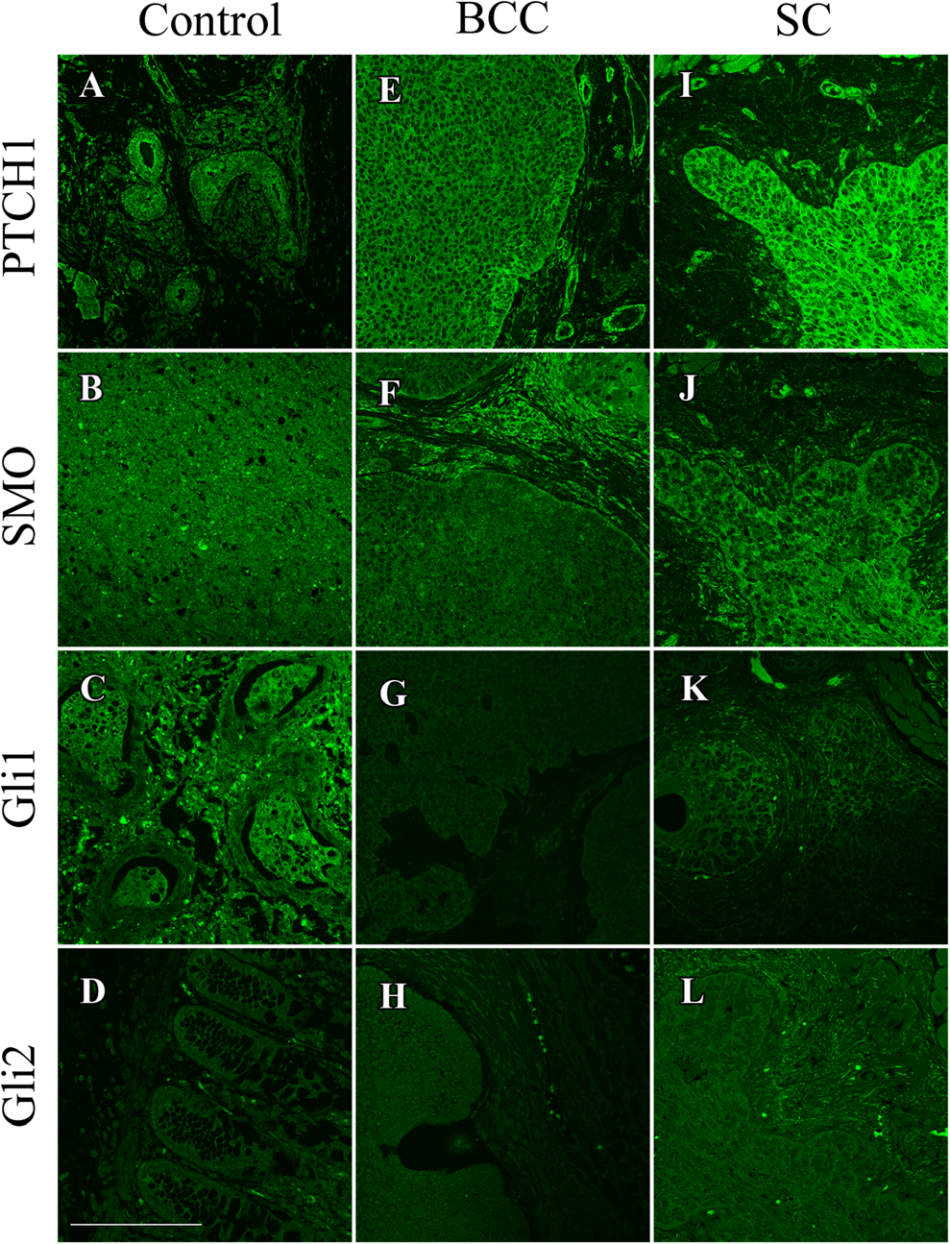
Representative pictures of Hedgehog pathway expression proteins using immunofluorescence in positive control tissue (**a** breast; **b** brain; **c** testicular; **d** intestinal), nBCC (**e**-**h**) and SC (**i**-**l**). Secondary antibody staining utilized AlexaFluor-568 and the color converted into black and white using ImageJ for pictorial purposes.* Scale bar* represents 250 μm and all images are at 200× magnification. Antibody stains for PTCH1 (**a**,** e**, **i**), SMO (**b**, **f**, **j**), Gli1 (**c**, **g**, **k**) and Gli2 (**d**,** h**,** l**).* PTCH1* Patched 1,* SMO* Smoothened,* Gli1* glioma-associated zinc transcription factor1,* Gli2* glioma-associated zinc transcription factor2

**Fig. 4:**
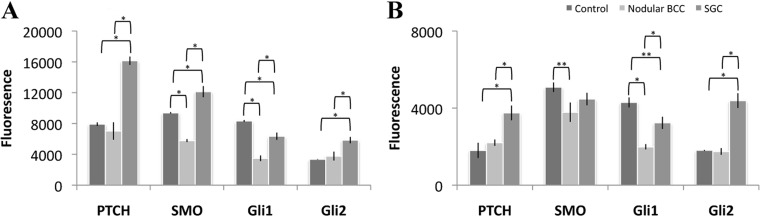
Semi-quantification of antibody expression (*x*-axis) within **a** SC tumor, nBCC tumor, control tissue and **b** stroma of SC, nBCC, control tissue using fluorescence intensity (*y*-axis) was determined in regions of interest as delineated by microscopy, ensuring a standardized area size whilst containing the same number of nuclei as determined by DAPI staining. Each tumor sample had an average of three readings. Each* bar* represents mean values ± SEM taken from 15 nBCC, 15 SC and control tissue samples for each Hh protein. *P < 0.01** *P* < 0.05.* PTCH1* Patched 1,* SMO* Smoothened,* Gli1* glioma-associated zinc transcription factor1,* Gli2* glioma-associated zinc transcription factor2

Immunofluorescence analysis was used to better quantify the amount of PTCH1, SMO, Gli1, and Gli2 expression in SC and nBCC (Fig. 3). A comparison between SC tumor and nBCC tumor was made along with physiologically activated Hh signaling (Fig. 4). PTCH1, SMO, Gli1, and Gli2 displayed higher levels of fluorescence in SC compared to nBCC (*P* < 0.01) (Fig. 4a). PTCH1, SMO, and Gli-2 were also expressed at higher levels in SC than physiological Hh signaling (*P* < 0.01). nBCC had similar levels of PTCH1 and Gli2 to physiologically activated Hh, but markedly lower expression of SMO and Gli1 (*P* < 0.01). Gli1 expression is lower in both tumors compared to normal expression; however, expression is still higher in SC than nBCC (*P* < 0.01).

Comparison of stromal expression using immunofluorescence (Fig. 4b) showed that PTCH1, Gli1, and Gli2 were more highly expressed in the stroma of SC compared to nBCC (*P* < 0.01). PTCH1 and Gli2 were highly upregulated in SC stroma compared to both normal expression and nBCC (*P* < 0.01). Gli1 is less expressed in SC (*P* < 0.05) and nBCC (*P* < 0.01) compared to physiological Hh expression.

## Discussion

This is the first study, as far as we are aware, that demonstrates the expression of the canonical Hh pathway, namely PTCH1, SMO, Gli1, and Gli2 in SC. Our results confirm that expression patterns are beyond normal reactivation levels, suggesting aberrant signaling.

DAB immunostaining is good for the detection of protein, however, quantification is highly subjective, for example, the use of the immunoreactivity score demonstrates significant interobserver variability has been shown in several studies [31, 32]. Immunofluorescence was employed to semi-quantify the amount of protein expression in the SC tumor in order to reduce this observer bias by computational counting along with the removal of background noise. Nevertheless, any immunohistochemistry method has many variables during processing, so relative expression to another tumor processed in an identical fashion is a more robust way of analyzing overall expression. In order to do this, a known aberrant Hh pathway tumor, nBCC, was selected for comparison [33]. Hh signaling is normally switched off in the majority of adult tissues, however, some do transiently express Hh. These normal tissue samples represent physiologically expressed Hh and were also used for comparison to determine if expression was simply re-activation or aberrant overexpression.

Levels of expression within SC tumor were significantly higher than those observed in nBCC. Furthermore, these levels were more than physiological re-activation, except for Gli1, which was less than in normal Hh-expressing tissue. It is estimated that up to 25% of tumors depend on the Hh pathway activity for growth [34]. Nodular BCC is a slow-growing cancer that metastasizes rarely [33]. In contrast, SC is locally aggressive, metastasizes and has a risk of mortality of up to 29% [8–10]. Thus, it is possible that the increased Hh expression seen in SC accounts for its more aggressive nature compared to nBCC. The exact role of Hh expression in SC cannot be discerned from this study alone, as Hh signaling has been implicated in tumorigenesis, growth, progression, local invasion, and metastasis [17–20].

The stroma of SC was found to express components of the Hh pathway, and in particular, Gli2 was considerably more upregulated. Cancer can modify the surrounding non-malignant tissue to facilitate local invasion [26]. Gli2 is an activator of the Hh pathway and plays a critical role in medulloblastoma tumorigenesis, with loss of Gli2 expression preventing the tumor formation [35, 36]. Moreover, higher levels of Gli2 are associated with a loss of the cell adhesion molecule E-cadherin in melanoma cell lines with increased capacity for local invasion; higher levels correlated with the most aggressive tumors [37, 38]. In contrast, knockdown models of Gli2 demonstrated reduced tumor growth in prostatic in vivo models [39]. Hh expression as a lone stimulator is unlikely and crosstalk with multiple pathways can occur. For example, transforming growth factor-β (TGF-β) has been shown to promote Gli2 expression and is involved in tumor progression and metastasis [40].

Activation of the Hh pathway in SC raises the possibility of medical treatment for these tumors, and potentially avoiding exenteration. Attenuation of the Hh pathway has been shown to be successful by using SMO inhibitors, with vismodegib presently being the most widely used compound in clinical trials and used for locally advanced or metastatic BCC [41, 42]. Furthermore, neoadjuvant treatment with vismodegib to reduce tumor size in BCC [43] has been successful in making the tumor more amenable to surgery. Vismodegib has been trialed in a variety of cancers, including pancreatic, ovarian cancer, breast and prostate cancers, and other SMO inhibitors are emerging such as sonidegib and BMS-833923 [44]. Gli antagonists have been developed too, and this may be a more promising level to block the pathway, especially Gli2 and could be used in combination to either block Hh at multiple levels or activated pathways [45]. It is possible that these agents could be efficacious in advanced or metastatic SC and if such a neoadjuvant response could be replicated, it may allow the preservation of sight by averting the need for a blinding exenteration.

Limitations of the study include a small sample size, although as SC is a rare tumor in Western countries, it is difficult to obtain high numbers. Further studies are required to identify the role of each protein in relation to SC behavior and could include tumorigenesis, propagation, local invasion, migration, or metastasis.

Hedgehog pathway activation occurs in periocular SC, in both the tumor and surrounding stromal tissue at a higher level than nBCC, a known Hh-driven tumor. The timing for any treatment modification of the Hh pathway would be dependent on its specific role in the pathogenesis of SC, and further studies are required to determine this.

## Electronic supplementary material

Supplementary Fig. S1 Eyelid lesions (A) right upper eyelid chalazion (B) Left upper eyelid sebaceous cell carcinoma and (C) right upper eyelid early chalazion (GIF 360 kb)

High resolution image (TIFF 1318 kb)

Supplementary Table S1 (PDF 20 kb)

Supplementary Table S2 (PDF 52 kb)

## References

[1] Deprez M, Uffer S (2009). Clinicopathological features of eyelid skin tumors. A retrospective study of 5504 cases and review of literature. Am J Dermatopathol.

[2] Kuzel P, Metelitsa AI, Dover DC, Salopek TG (2012). Epidemiology of sebaceous carcinoma in Alberta, Canada, from 1988 to 2007. J Cutan Med Surg.

[3] Xu XL, Li B, Sun XL, Li LQ, Ren RJ, Gao F, Jonas JB (2008). Eyelid neoplasms in the Beijing Tongren eye Centre between 1997 and 2006. Ophthalmic Surg Lasers Imaging: Off J Int Soc Imaging Eye.

[4] Muqit MM, Foot B, Walters SJ, Mudhar HS, Roberts F, Rennie IG (2013). Observational prospective cohort study of patients with newly-diagnosed ocular sebaceous carcinoma. Br J Ophthalmol.

[5] Obata H, Aoki Y, Kubota S, Kanai N, Tsuru T (2005). Incidence of benign and malignant lesions of eyelid and conjunctival tumors. Nippon Ganka Gakkai Zasshi.

[6] Chao AN, Shields CL, Krema H, Shields JA (2001). Outcome of patients with periocular sebaceous gland carcinoma with and without conjunctival intraepithelial invasion. Ophthalmology.

[7] Buitrago W, Joseph AK (2008). Sebaceous carcinoma: the great masquerader: emerging concepts in diagnosis and treatment. Dermatol Ther.

[8] Doxanas MT, Green WR (1984). Sebaceous gland carcinoma. Review of 40 cases. Arch Ophthalmol.

[9] Shields JA, Demirci H, Marr BP, Eagle RC, Shields CL (2004). Sebaceous carcinoma of the eyelids: personal experience with 60 cases. Ophthalmology.

[10] Ni C, Searl SS, Kuo P, Cho F, Chong C, Albert D (1982). Sebaceous cell carcinomas of the ocular adnexa. Int Ophthalomol Clin.

[11] Dogru M, Matsuo H, Inoue M, Okubo K, Yamamoto M (1997). Management of eyelid sebaceous carcinomas. Ophthalmol J Int d'Ophtal Int J Ophthalmol Z Augenheil.

[12] Song A, Carter KD, Syed NA, Song J, Nerad JA (2008). Sebaceous cell carcinoma of the ocular adnexa: clinical presentations, histopathology, and outcomes. Ophthal Plast Reconstr Surg.

[13] Petrova R, Joyner AL (2014) Roles for Hedgehog signaling in adult organ homeostasis and repair. Development (Cambridge, England). 2014. Published by The Company of Biologists Ltd., England, pp. 3445–3457 10.1242/dev.083691 PMC4197719 25183867

[14] Sahin Z, Szczepny A, McLaughlin EA, Meistrich ML, Zhou W, Ustunel I, Loveland KL (2014). Dynamic hedgehog signalling pathway activity in germline stem cells. Andrology.

[15] Ruiz i Altaba A, Sanchez P, Dahmane N (2002). Gli and hedgehog in cancer: tumours, embryos and stem cells. Nat Rev Cancer.

[16] Berman DM, Karhadkar SS, Maitra A, Montes De Oca R, Gerstenblith MR, Briggs K, Parker AR, Shimada Y, Eshleman JR, Watkins DN, Beachy PA (2003). Widespread requirement for hedgehog ligand stimulation in growth of digestive tract tumours. Nature.

[17] Watkins DN, Berman DM, Baylin SB (2003). Hedgehog signaling: progenitor phenotype in small-cell lung cancer. Cell Cycle (Georgetown, Tex).

[18] Thayer SP, di Magliano MP, Heiser PW, Nielsen CM, Roberts DJ, Lauwers GY, Qi YP, Gysin S, Fernandez-del Castillo C, Yajnik V, Antoniu B, McMahon M, Warshaw AL, Hebrok M (2003). Hedgehog is an early and late mediator of pancreatic cancer tumorigenesis. Nature.

[19] Dahmane N, Lee J, Robins P, Heller P, Ruiz i Altaba A (1997). Activation of the transcription factor Gli1 and the sonic hedgehog signalling pathway in skin tumours. Nature.

[20] Sanchez P, Clement V, Ruiz i Altaba A (2005). Therapeutic targeting of the hedgehog-GLI pathway in prostate cancer. Cancer Res.

[21] Ng JM, Curran T (2011). The Hedgehog's tale: developing strategies for targeting cancer. Nat Rev Cancer.

[22] Davidson B, Trope CG, Reich R (2014). The role of the tumor stroma in ovarian cancer. Front Oncol.

[23] Podlasek CA, Barnett DH, Clemens JQ, Bak PM, Bushman W (1999). Prostate development requires sonic hedgehog expressed by the urogenital sinus epithelium. Dev Biol.

[24] Wilkinson SE, Furic L, Buchanan G, Larsson O, Pedersen J, Frydenberg M, Risbridger GP, Taylor RA (2013). Hedgehog signaling is active in human prostate cancer stroma and regulates proliferation and differentiation of adjacent epithelium. Prostate.

[25] Kataoka F, Tsuda H, Arao T, Nishimura S, Tanaka H, Nomura H, Chiyoda T, Hirasawa A, Akahane T, Nishio H, Nishio K, Aoki D (2012). EGRI and FOSB gene expressions in cancer stroma are independent prognostic indicators for epithelial ovarian cancer receiving standard therapy. Genes Chromosom Cancer.

[26] Marsh D, Dickinson S, Neill GW, Marshall JF, Hart IR, Thomas GJ (2008). Alpha vbeta 6 integrin promotes the invasion of morphoeic basal cell carcinoma through stromal modulation. Cancer Res.

[27] Slater DW, M (2014) Standards and datasets for reporting cancers: Dataset for the histological reporting of primary cutaneous basal cell carcinoma. https://www.rcpath.org/resourceLibrary/g123-data-set-basal-may-2014.html. Accessed 4 Oct 2017

[28] Slater DWM (2014) Standards and datasets for reporting cancers: Dataset for the histological reporting of primary cutaneous adnexal carcinomas and regional lymph nodes. https://www.rcpath.org/resourceLibrary/dataset-for-the-histological-reporting-of-primary-cutaneous-adnexal-carcinomas-and-regional-lymph-nodes-.html. Accessed 4 Oct 2017

[29] Badve S, Vladislav IT, Spaulding B, Strickland A, Hernandez S, Bird-Turner L, Dodson C, Elleby B, Phillips T (2013). EP1: a novel rabbit monoclonal antibody for detection of oestrogen receptor alpha. J Clin Pathol.

[30] Nadendla SK, Hazan A, Ward M, Harper LJ, Moutasim K, Bianchi LS, Naase M, Ghali L, Thomas GJ, Prowse DM, Philpott MP, Neill GW (2011). GLI1 confers profound phenotypic changes upon LNCaP prostate cancer cells that include the acquisition of a hormone independent state. PLoS One.

[31] Parker RL, Huntsman DG, Lesack DW, Cupples JB, Grant DR, Akbari M, Gilks CB (2002). Assessment of interlaboratory variation in the immunohistochemical determination of estrogen receptor status using a breast cancer tissue microarray. Am J Clin Pathol.

[32] von Wasielewski R, Mengel M, Wiese B, Rudiger T, Muller-Hermelink HK, Kreipe H (2002). Tissue array technology for testing interlaboratory and interobserver reproducibility of immunohistochemical estrogen receptor analysis in a large multicenter trial. Am J Clin Pathol.

[33] Saldanha G, Fletcher A, Slater DN (2003). Basal cell carcinoma: a dermatopathological and molecular biological update. Br J Dermatol.

[34] Lum L, Beachy PA (2004). The hedgehog response network: sensors, switches, and routers. Sci.

[35] Hui CC, Angers S (2011). Gli proteins in development and disease. Annu Rev Cell Dev Biol.

[36] Flora A, Klisch TJ, Schuster G, Zoghbi HY (2009). Deletion of Atoh1 disrupts sonic hedgehog signaling in the developing cerebellum and prevents medulloblastoma. Sci.

[37] Alexaki VI, Javelaud D, Van Kempen LC, Mohammad KS, Dennler S, Luciani F, Hoek KS, Juarez P, Goydos JS, Fournier PJ, Sibon C, Bertolotto C, Verrecchia F, Saule S, Delmas V, Ballotti R, Larue L, Saiag P, Guise TA, Mauviel A (2010). GLI2-mediated melanoma invasion and metastasis. J Natl Cancer Inst U S.

[38] Pantazi E, Gemenetzidis E, Teh MT, Reddy SV, Warnes G, Evagora C, Trigiante G, Philpott MP (2017) GLI2 is a regulator of beta-catenin and is associated with loss of E-cadherin, cell invasiveness, and long-term epidermal regeneration. J Invest Dermatol. 10.1016/j.jid.2016.11.046 10.1016/j.jid.2016.11.046 28300597

[39] Thiyagarajan S, Bhatia N, Reagan-Shaw S, Cozma D, Thomas-Tikhonenko A, Ahmad N, Spiegelman VS (2007). Role of GLI2 transcription factor in growth and tumorigenicity of prostate cells. Cancer Res U S.

[40] Javelaud D, Alexaki VI, Dennler S, Mohammad KS, Guise TA, Mauviel A (2011). TGF-beta/SMAD/GLI2 signaling axis in cancer progression and metastasis. Cancer Res.

[41] LoRusso PM, Rudin CM, Reddy JC, Tibes R, Weiss GJ, Borad MJ, Hann CL, Brahmer JR, Chang I, Darbonne WC, Graham RA, Zerivitz KL, Low JA, Von Hoff DD (2011). Phase I trial of hedgehog pathway inhibitor vismodegib (GDC-0449) in patients with refractory, locally advanced or metastatic solid tumors. Clin Cancer Res.

[42] Demirci H, Worden F, Nelson CC, Elner VM, Kahana A (2015) Efficacy of Vismodegib (Erivedge) for basal cell carcinoma involving the orbit and periocular area. Ophthal Plast Reconstr Surg. 10.1097/iop.0000000000000388 10.1097/IOP.0000000000000388 PMC4564370 25675162

[43] Ally MS, Aasi S, Wysong A, Teng C, Anderson E, Bailey-Healy I, Oro A, Kim J, Chang AL, Tang JY (2014). An investigator-initiated open-label clinical trial of vismodegib as a neoadjuvant to surgery for high-risk basal cell carcinoma. J Am Acad Dermatol.

[44] Queiroz KC, Spek CA, Peppelenbosch MP (2012). Targeting hedgehog signaling and understanding refractory response to treatment with hedgehog pathway inhibitors. Drug Resist Updat.

[45] Latuske EM, Stamm H, Klokow M, Vohwinkel G, Muschhammer J, Bokemeyer C, Jucker M, Kebenko M, Fiedler W, Wellbrock J (2017). Combined inhibition of GLI and FLT3 signaling leads to effective anti-leukemic effects in human acute myeloid leukemia. Oncotarget.

