# Supplementary figures and images for: Analysis of hedgehog signaling in periocular sebaceous carcinoma
Source: Graefes Arch Clin Exp Ophthalmol. 2018 Feb 8;256(4):853–60. doi: 10.1007/s00417-018-3900-5 (PMC5856882; doi:10.1007/s00417-018-3900-5)

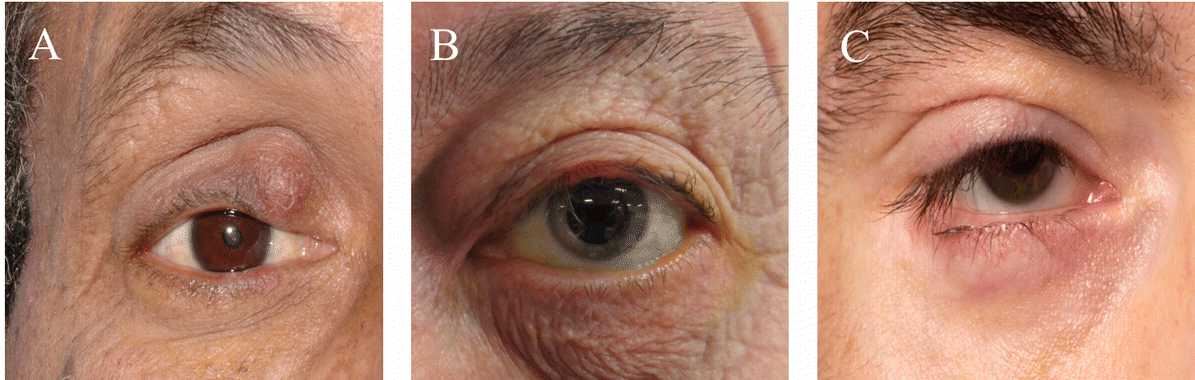

Supplement: Supplementary file 1 — Eyelid lesions (A) right upper eyelid chalazion (B) Left upper eyelid sebaceous cell carcinoma and (C) right upper eyelid early chalazion (GIF 360 kb) [file 417_2018_3900_Fig5_ESM.gif]

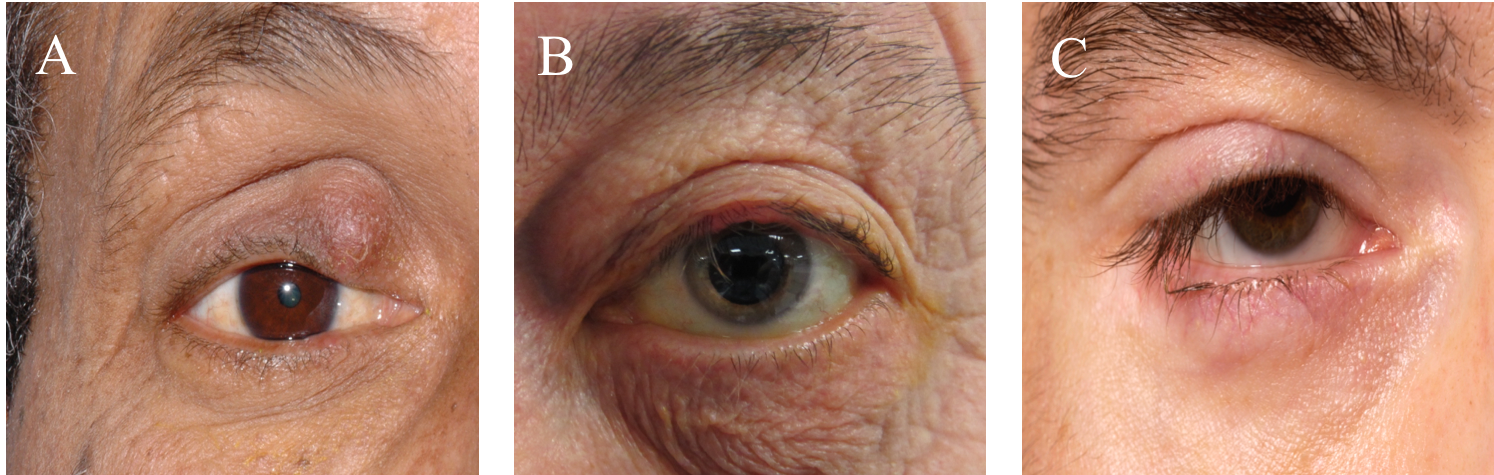

Supplement: Supplementary file 2 — High resolution image (TIFF 1318 kb) [file 417_2018_3900_MOESM1_ESM.tif]
