# Supplementary material for: Analysis of hedgehog signaling in periocular sebaceous carcinoma
Source: Graefes Arch Clin Exp Ophthalmol. 2018 Feb 8;256(4):853–60. doi: 10.1007/s00417-018-3900-5 (PMC5856882; doi:10.1007/s00417-018-3900-5)
Supplement: Supplementary file 3 — (PDF 20 kb) [file 417_2018_3900_MOESM2_ESM.pdf]

| Cancer | Hh pathway signalling | Reference |
|---|---|---|
| BCC | 90% | [1] |
| Medulloblastoma | 30% | [2] |
| Small cell lung cancer | 25% | [3] |
| Prostate | Involved | [4] |
| Glioma | Involved | [5] |

Supplementary Table S2. Percentage of involvement of aberrant hedgehog (Hh) signaling pathway in different solid tumours.


1. Ng JM, Curran T (2011) The Hedgehog's tale: developing strategies for targeting cancer. Nature reviews Cancer 11: 493-501 DOI 10.1038/nrc3079
2. Kool M, Koster J, Bunt J, Hasselt NE, Lakeman A, van Sluis P, Troost D, Meeteren NS, Caron HN, Cloos J, Mrsic A, Ylstra B, Grajkowska W, Hartmann W, Pietsch T, Ellison D, Clifford SC, Versteeg R (2008) Integrated genomics identifies five medulloblastoma subtypes with distinct genetic profiles, pathway signatures and clinicopathological features. PLoS One 3: e3088 DOI 10.1371/journal.pone.0003088
3. Cohen DJ (2012) Targeting the hedgehog pathway: role in cancer and clinical implications of its inhibitionHematol Oncol Clin North Am. 2012 Elsevier Inc, United States, pp. 565-588, viii.
4. Abate-Shen C, Shen MM (2000) Molecular genetics of prostate cancer. Genes & development 14: 2410-2434
5. Gu W, Shou J, Gu S, Sun B, Che X (2014) Identifying Hedgehog Signaling Specific MicroRNAs in GlioblastomasInt J Med Sci, pp. 488-493.
