# Supplementary material for: Analysis of hedgehog signaling in periocular sebaceous carcinoma
Source: Graefes Arch Clin Exp Ophthalmol. 2018 Feb 8;256(4):853–60. doi: 10.1007/s00417-018-3900-5 (PMC5856882; doi:10.1007/s00417-018-3900-5)
Supplement: Supplementary file 4 — (PDF 52 kb) [file 417_2018_3900_MOESM3_ESM.pdf]

Antibody	Concentration	Ag retrieval
PTCH<br>Ab39266	1 in 1500	High pH 9<br>20 mins 97°C
SMO<br>Ab72130	1 in 200	High pH 9<br>20 mins 97°C
Gli1<br>Ab134906	1 in 250	High pH 9<br>20 mins 97°C
Gli2<br>Ab26056	1 in 150	Low pH 6<br>20 mins 97°C
Alexa Fluro® 568<br>A11011	1 in 500	-

Table S1 Summary of antibodies used for immunohistochemistry. Antigen retrieval used the DAKO PT LINK (Dako, Glostrup, Denmark) machine at 97°C for 20 minutes under alkaline (pH 9.0) or acidic (pH 6.0) conditions. Ab= Abcam, Cambridge, UK; A= Thermo Fisher Scientific Inc, Waltham, MA, USA; Ag=Antigen; PTCH1=Patched 1; SMO=smoothened; Gli1=Glioma-associated zinc transcription factor1; Gli2= Glioma-associated zinc transcription factor2=Gli2
